# Defective Proliferation and Osteogenic Potential with Altered Immunoregulatory phenotype of Native Bone marrow-Multipotential Stromal Cells in Atrophic Fracture Non-Union

**DOI:** 10.1038/s41598-019-53927-3

**Published:** 2019-11-22

**Authors:** Jehan J. El-Jawhari, George Kleftouris, Yasser El-Sherbiny, Hany Saleeb, Robert M. West, Elena Jones, Peter V. Giannoudis

**Affiliations:** 1 Leeds Institute of Rheumatic and Musculoskeletal Medicine, School of Medicine, University of Leeds, Leeds, UK; 2 NIHR Leeds Biomedical Research Centre, Chapel Allerton Hospital, Leeds, UK; 3 Clinical pathology department, Mansoura University, Mansoura, Egypt; 4 Academic Department of Trauma and Orthopaedic, Leeds General Infirmary, School of Medicine, University of Leeds, Leeds, UK; 5 Department of Biosciences, School of Science and Technology, Nottingham Trent University, Nottingham, UK; 6 Leeds Institute of Health Sciences, University of Leeds, Leeds, UK

**Keywords:** Osteoimmunology, Multipotent stem cells, Regeneration

## Abstract

Bone marrow-Multipotential stromal cells (BM-MSCs) are increasingly used to treat complicated fracture healing e.g., non-union. Though, the quality of these autologous cells is not well characterized. We aimed to evaluate bone healing-related capacities of non-union BM-MSCs. Iliac crest-BM was aspirated from long-bone fracture patients with normal healing (U) or non-united (NU). Uncultured (native) CD271highCD45low cells or passage-zero cultured BM-MSCs were analyzed for gene expression levels, and functional assays were conducted using culture-expanded BM-MSCs. Blood samples were analyzed for serum cytokine levels. Uncultured NU-CD271highCD45low cells significantly expressed fewer transcripts of growth factor receptors, EGFR, FGFR1, and FGRF2 than U cells. Significant fewer transcripts of alkaline phosphatase (ALPL), osteocalcin (BGLAP), osteonectin (SPARC) and osteopontin (SPP1) were detected in NU-CD271^high^CD45^low^ cells. Additionally, immunoregulation-related markers were differentially expressed between NU- and U-CD271^high^CD45^low^ cells. Interestingly, passage-zero NU BM-MSCs showed low expression of immunosuppressive mediators. However, culture-expanded NU and U BM-MSCs exhibited comparable proliferation, osteogenesis, and immunosuppression. Serum cytokine levels were found similar for NU and U groups. Collectively, native NU-BM-MSCs seemed to have low proliferative and osteogenic capacities; therefore, enhancing their quality should be considered for regenerative therapies. Further research on distorted immunoregulatory molecules expression in BM-MSCs could potentially benefit the prediction of complicated fracture healing.

## Introduction

Despite advances in trauma fixation techniques within the orthopaedic field, atrophic fracture non-union is still significant health and socioeconomic burden^1–5^. Multipotential stromal cells (BM-MSCs) have been acknowledged for enhancing bone healing particularly in complicated healing conditions such as atrophic fracture non-union^6–10^. However, autologous native BM-MSCs are usually applied as a part of bone marrow (BM) aspirates or concentrate without an assessment of the quality of these cells. Furthermore, inadequate research data on the quality of these potential therapeutic cells has led to a lack of consensus on whether complementary growth adjuvants are required to enhance their healing capacities.

MSCs can migrate from BM and other adjacent tissues within a few days of bone injury, and are highly involved in the three phases of the healing process; inflammatory, repair and remodelling^11–14^. During the inflammatory phase, a hematoma is formed involving several immune cells and mediators^12,13,15–18^. However, a balanced immune response is required for successful bone healing. A clinical removal of fracture hematoma in elderly or multi-diseased patients is perceived as a cause delayed or impaired healing^19^. Additionally, complicated healing was strongly inked to prolonged local inflammatory response^20–22^. The presence of MSCs within the fracture hematoma has been reported^12^ with an indication of their involvement in immunosuppression to end the inflammatory phase and to start bone repair^23^. Furthermore, applying MSCs has been linked to decrease the levels of inflammatory cytokines in association with enhancing the osteogenesis in experimental models of fractures and osteoporosis^24–26^. However, only MSCs primed by interferon gamma (IFN-γ), tumour necrosis factor-alpha (TNF-α), interleukin-1 (IL-1), and interleukin-17 (IL-17) can produce immunosuppressive factors such as Tumour Growth Factor-beta 1 (TGF-β1), Prostaglandin E2 (PGE2) and Indoleamine 2,3-Dioxygenase (IDO)^23^.

MSC differentiation into osteoblasts is also essential for bone repair and remodelling. The osteogenic capacity of MSCs typically starts with proliferation and expression of specific proteins, e.g., osteopontin (SPP1), followed by matrix maturation with alkaline phosphatase (ALP) expression. Finally, mineralisation is characterised by increased ALP and osteocalcin (BGLAP) expression^27^. Furthermore, MSCs support new bone vasculature via Vascular Endothelial Growth Factor (VEGF),^28^. Recently, Bone Marrow Stromal Cell Antigen-2 (BST2) was identified as a marker for some BM-MSC clones with immunoregulatory capacities^29^. Furthermore, BST2 knockdown in BM-MSCs has revealed its role in osteogenic differentiation through the regulation of the bone morphogenic protein 2 (BMP-2) signalling pathway^30^. Additionally, another protein related to mineralisation and highly expressed in osteoblasts, S100 calcium-binding protein A8 (S100A8) was linked to MSC-mediated immunoregulation^31^, but with no link to the MSC osteogenic capacity.

Several studies reported biological mechanisms underlying fracture atrophic non-union development with the involvement of the osteogenesis-related BMP and Wnt signalling pathways^32–36^. However, the research examining MSC biology in non-union fracture has been focused on their presence and quantities. These osteoprogenitors were detected within the tissues of non-union as expressing stem cell markers^36–38^. Noteworthy, lower numbers of BM-derived colony-forming cells were reported for atrophic non-union than healed fractures patients^39^; however, no difference was found in BM-MSC numbers expressing osteoprogenitor marker, CD271^high^ in another study^40^. Other studies have reported a similar osteogenic function of MSCs resident within non-union tissues relative to donor-matched BM-MSCs but without comparison to union-MSCs^36,37,41,42^. Furthermore, the immunoregulatory potential of non-union BM-MSCs is mostly unknown despite being an important part of the bone healing process.

We aimed in this study to investigate bone healing-related functions of BM-MSCs in non-united fractures. Consequently, we compared proliferation, osteogenesis, and immunoregulation of BM-MSCs from patients who had established non-union fractures (NU) with those from patients with united fractures (U). These BM-MSCs were analysed either without culture as CD271^high^ CD45^low^ cells, the surrogates of BM-MSCs^43–46^, or with a minimal culture (passage-zero cells). Culture-expansion of these BM-MSCs as standardly characterised^47^ was conducted to generate sufficient cell numbers for the functional assays. To investigate the potential systemic/serum influence on NU BM-MSC functions, we assessed the serum levels of priming cytokines and their receptor expression in BM-MSCs. This knowledge could help to further understand the biological role of MSCs during the development of complicated bone healing and potentially aid to optimise the autologous BM-MSC based regenerative therapies.

## Results

### The isolation and characterisation of uncultured BM-cells

**Figure 1:**
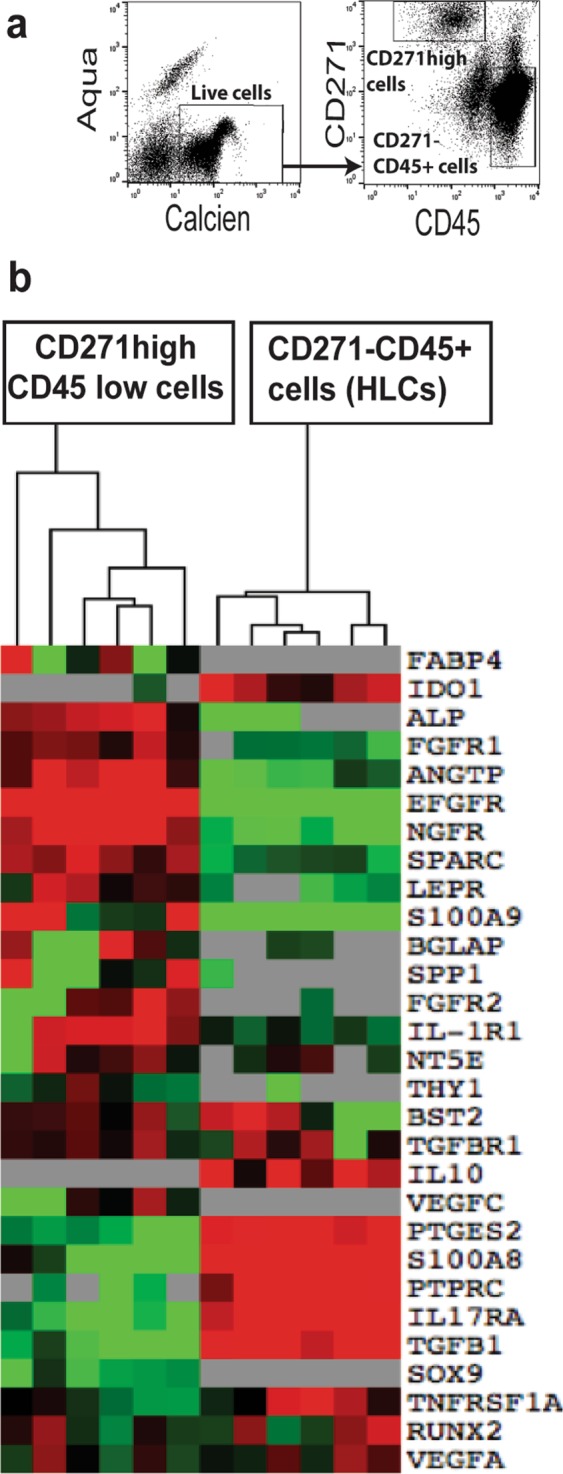
The isolation of uncultured BM CD271^high^ CD45^low^ cells. (**a**) The FACS plots show the gating strategy for isolation of live BM cells (as Aqua ^negative^ Calcein ^positive^) then uncultured BM-MSCs as CD271^high^ CD45^low^ and HLCs (CD45^positive^ CD271^negative^). (**b**) The clustering of donor-matched CD271^high^ CD45^low^ and HLCs (n = 6) for expression of growth, osteogenic, and immune-related markers. Green: low expression. Red: high expression. Grey: below detection.

To test the functional markers of uncultured (native) BM-MSCs, the FACS isolation of BM-cells (both NU and U) was conducted. The live cells were marked as Calcein^positive^ Aqua^negative^ cells, then CD271^high^ CD45^low^ (MSC surrogates) and CD271^negative^ CD45^positive^ cells hematopoietic lineage cells (HLCs) were isolated for gene expression assays (Fig. 1a). Distinct differences between CD271^high^ CD45^low^ cells and HLCs were evident as shown via the clustering of transcripts levels and comparative group analysis (Fig. 1b and Table 1). As expected, the transcript levels of *NGFR* (CD271) and osteogenic markers, *BGLAP, ALPL, SPARC, LepR and SPP1* were higher for CD271^high^ CD45^low^ cells than HLCs (p < 0.0001). Uniquely, more transcripts of *IL-1R1* (p = 0.003), Fibroblast growth factor receptors 1 (*FGFR1*), and epidermal growth factor receptor (*EGFR*) were also detected for CD271^high^ CD45^low^ cells (p < 0.001). However, *PTPRC* (CD45), *TGF-ß*, Prostaglandin E synthetase 2 *(PGES2), IL-17RA*, and *TNFRS1A* transcripts were significantly lower in CD271^high^ CD45^low^ cells than HLCs (p < 0.0001). Furthermore, *IDO* and *IL-10* transcripts were not detected in CD271^high^ CD45^low^ cells. Collectively, uncultured sorted CD271^high^ CD45^low^ cells showed specific MSC gene profile compared to HLCs. Based on these findings, we consequently assessed the functional markers for sorted CD271^high^ CD45^low^ cells.

**Table 1:** TaqMan markers used in the study and gene expression differences between groups.

Functions	Gene	TaqMan assay	Full name	CD271^high^ cells versus CD45^+^ cells (HLCs)	NU CD271^high^ cells versus U CD271^high^ cells
				Fold	Fold
Surface cell markers	*NGFR*	HS00609977_m1	Nerve growth factor receptor (CD271)	1100 (+)*	3.1 (−)*
	*PTPRC*	HS00894734_m1	Protein Tyrosine Phosphatase Receptor Type C (CD45)	6.5 (−)*	ND
	*NT5E*	Hs00159686_m1	5′-Nucleotidase Ecto (CD73)	40.5 (+)*	1.5 (−)
	*THY1*	Hs00174816-m1	Thymus-like antigen 1 (CD90)	53.3 (+)*	2.3 (−)
Growth Factor Receptors	*FGFR1*	Hs00241111_m1	Fibroblast growth factor receptor 1	108 (+)*	2.2 (−)*
	*FGFR2*	Hs01552926_m1	Fibroblast growth factor receptor 2	ND	2.4 (−)*
	*EGFR*	Hs01076078_m1	Epidermal growth factor receptor	26 (+)*	3 (−)*
	*TGF-βR1*	HS00610319_m1	Transforming growth factor beta receptor 1	22 (+))*	1.4 (−)
Angiogenesis related	*VEGFA*	Hs00900058_m1	Vascular endothelial growth factor A	12.6 (+)*	1.5 (−)*
	*VEGFC*	Hs01099206_m1	Vascular endothelial growth factor C	ND	1.6 (+)
	*ANGPT1*	Hs00181613_m1	Angiopoietin 1	352 (+)*	1.33 (−)
Chondrogenic and Adipogenic Differentiation	*FABP4*	Hs00609791_m1	Fatty acid binding protein 4	ND	1.4 (+)
	*LepR*	Hs00174492_m1	Leptin Receptor, encoding CD295protein	197 (+)*	1.5 (+)
	*SOX9*	Hs00165814_m1	SRY (sex-determining region Y)-box 9	ND	1.5 (+)
Osteogenic Differentiation	*SPARC*	Hs00277762_m1	Secreted protein acidic and rich in cysteine	155.7 (+)*	6.3 (−)*
	*RUNX2*	Hs00231692_m1	Runt related transcription factor2	9 (+)*	1.4 (−)
	*ALPL*	Hs00758162_m1	Alkaline Phosphatase	2755 (+) *	2.8 (−)*
	*BGLAP*	Hs01587814_g1	Bone gamma-carboxy-glutamic acid-containing protein	ND	4.8 (−)*
	*SPP1*	Hs00959010_m1	Secreted phosphoprotein 1 (Osteopontin)	ND	5.3 (−)*
Cytokine receptors	*IL-1R1*	Hs00991010_m1	Interleukin-1 receptor 1	215 (+)*	2 (−)*
	*TNFRS1A*	Hs01042313_m1	TNF receptor superfamily member 1A (CD120a)	2.5 (−) *	1.1 (−)
	*IL-17RA*	Hs01056316_m1	Interleukin 17 receptor A (CD217)	3 (−) *	2 (−)
	*IFNGR1*	Hs00988304_m1	Interferon gamma receptor 1 (CD119)	1.1 (+)	1.4 (−)
Immunosuppressive Factors	*IDO*	Hs00984148_m1	Indoleamine 2,3-dioxygenase	ND	ND
	*IL-10*	Hs00961622_m1	Interleukin-10	ND	ND
	*PTGES2*	Hs00228159_m1	Prostaglandin E synthetase 2	2.3 (−)	1.7 (−)
	*TGF-β1*	Hs00998133_m1	Transforming growth factor beta 1	3 (−)*	1.2 (+)
Immune-related Factors	*S100A8*	Hs00374264_g1	S100 calcium-binding protein A8	14.5 (−)*	3.4 (+)*
	*S100A9*	Hs00610058_m1	S100 calcium-binding protein A9	90 (+)*	2.5 (−)
	*BST2*	Hs00171632_m1	Bone marrow stromal antigen 2	18.5 (+)*	2.8 (−)*
Housekeeping gene	*HPRT1*	Hs99999909_m1	Hypoxanthine Phosphoribosyl Transferase 1		

Comparison of CD271^high^ CD45^low^ cells versus CD45^positive^ CD271^negative^ cells (HLCs) was donor-matched (n = 6).

Comparison of NU CD271^high^ CD45^low^ cells versus U CD271^high^ CD45^low^ cells was not donor-matched (n = 8 and 9 respectively).

^*^Significant difference p < 0.05. (+): higher expression. (−): lower expression.

ND: not determined fold difference as the marker gene expression was below detection in one of the groups.

### A defective proliferation and osteogenic potential of uncultured and minimally-cultured BM-MSCs

**Figure 2:**
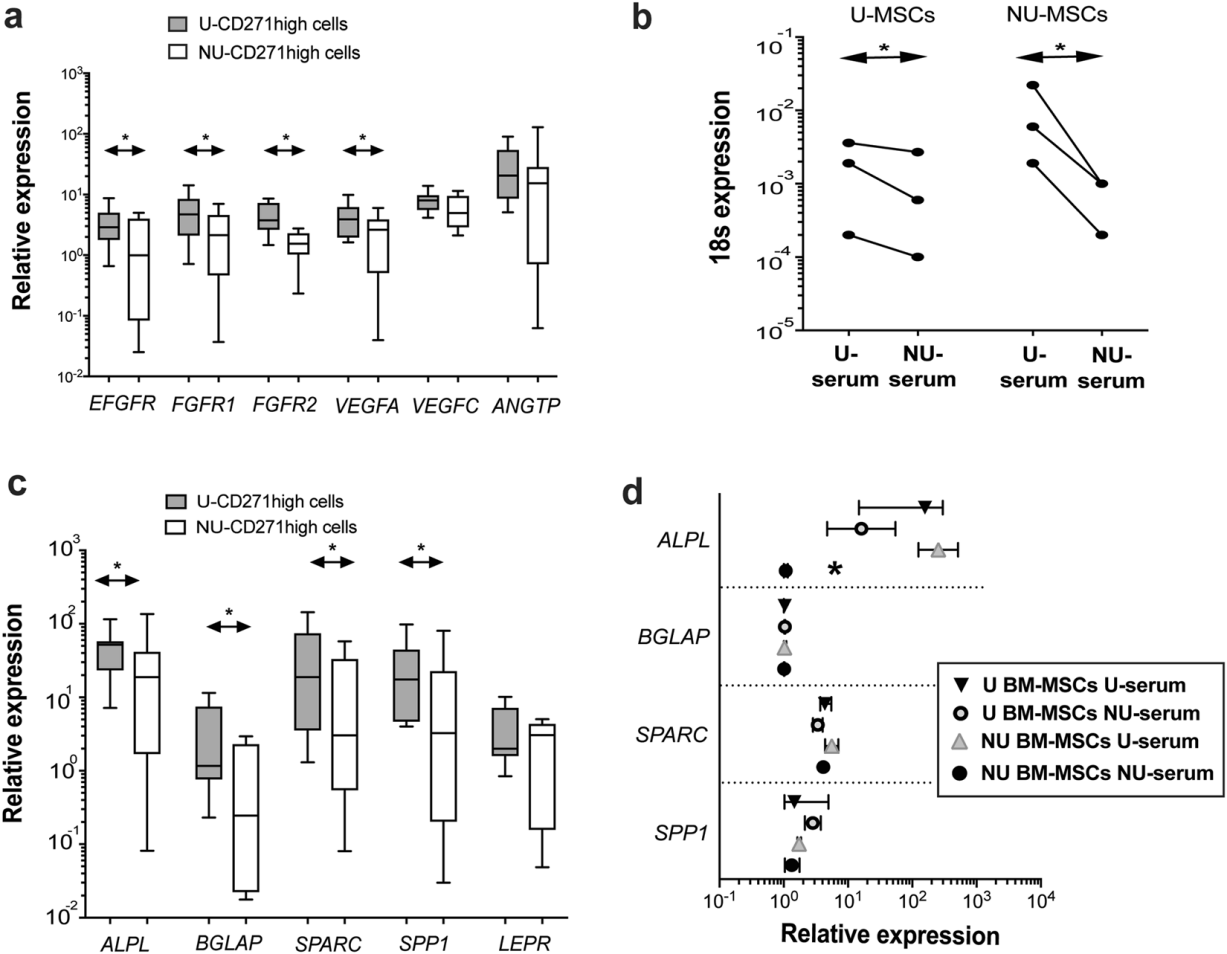
The proliferative potential and osteogenic markers of NU BM-MSCs. (**a**) The figure shows the median of relative gene expression of growth factors receptors and angiogenic factors. The unpaired t-test was used to compare uncultured isolated NU- CD271^high^ CD45^low^ cells (n = 8) and U-CD271^high^ CD45^low^ cells (n = 9). (**b**) The figure shows the relative gene expression of 18 s for passage-zero NU or U BM-MSCs in cultures supplemented with either U-serum or NU-serum. The Wilcoxon test was used to compare passage-zero either NU or U BM-MSCs (n = 3) between cultures supplemented with U-serum and NU-serum. (**c**) The figure shows the median of relative gene expression of osteogenic markers. The Mann Whitney test was used to compare uncultured isolated NU-CD271^high^ CD45^low^ cells (n = 8) and U-CD271^high^ CD45^low^ cells (n = 9). (**d**) The figure presents the median (with interquartile range) of relative gene expression of osteogenic markers for passage-zero BM-MSCs in either U-serum or NU-serum supplemented cultures. The Kruskal-Wallis test was used to compare the groups (n = 3).

Following the sorting of BM cells, the growth factor receptors and osteogenic markers were tested on isolated uncultured CD271^high^ CD45^low^ cells. Interestingly, fewer transcript levels of *EGFR, FGFR1* and *FGRF2* were detected in uncultured NU-CD271^high^ CD45^low^ than U-cells (p = 0.049, p = 0.021, and p < 0.001 respectively, Fig. 2a and Table 1) indicating potentially a low response to these growth factors. When measuring the growth factors related to angiogenesis, the *VEGFA* transcripts (but not *VEGFC* or *ANGTP*) were lower in NU-CD271^high^ CD45^low^ cells than U-cells (p = 0.032, Fig. 2a and Table 1). To test if serum has an impact (systemic) on the proliferation of BM-MSCs, BM cells were seeded in cultures with either NU- or U-serum then the levels of 18 s were assessed (without cell passage) as indicative of cell quantities. Noticeably, the donor-matched comparison showed that both NU- and U-passage-zero BM-MSCs had reduced 18 s levels when in NU serum cultures than those in U-serum cultures (p = 0.031 for both, Fig. 2b) indicating a negative effect of NU serum on MSC proliferation.

The PCR data showed significantly lower levels of osteogenic markers, *ALPL*, *BGLAP*, *SPARC* and *SPP1*, but not *LepR* in uncultured NU-CD271^high^ CD45^low^ cells than U-cells (p = 0.040, p = 0.049, p = 0.043 & p = 0.047 respectively, Fig. 2c and Table 1) suggesting an impaired osteogenic potential for NU BM-MSCs. When cultured in NU-serum, passage-zero NU BM-MSCs had consistently less *ALPL* transcripts relative to U-serum cultures (p = 0.008) and U-MSCs in both serum cultures (both p = 0.029, Fig. 2d). However, *BGLAP, SPP1* and *SPARC* transcripts were comparable in both U and NU serum-cultures (Fig. 2d). In summary, reduced the proliferative potential of NU BM-MSCs was noted, and apparently due to both cellular and serum-related causes. Also, osteogenic markers were less expressed on native BM-MSCs, but that was not as evident in serum-supplemented cultures.

### An altered immunoregulatory phenotype of uncultured and minimally-cultured NU BM-MSCs

**Figure 3:**
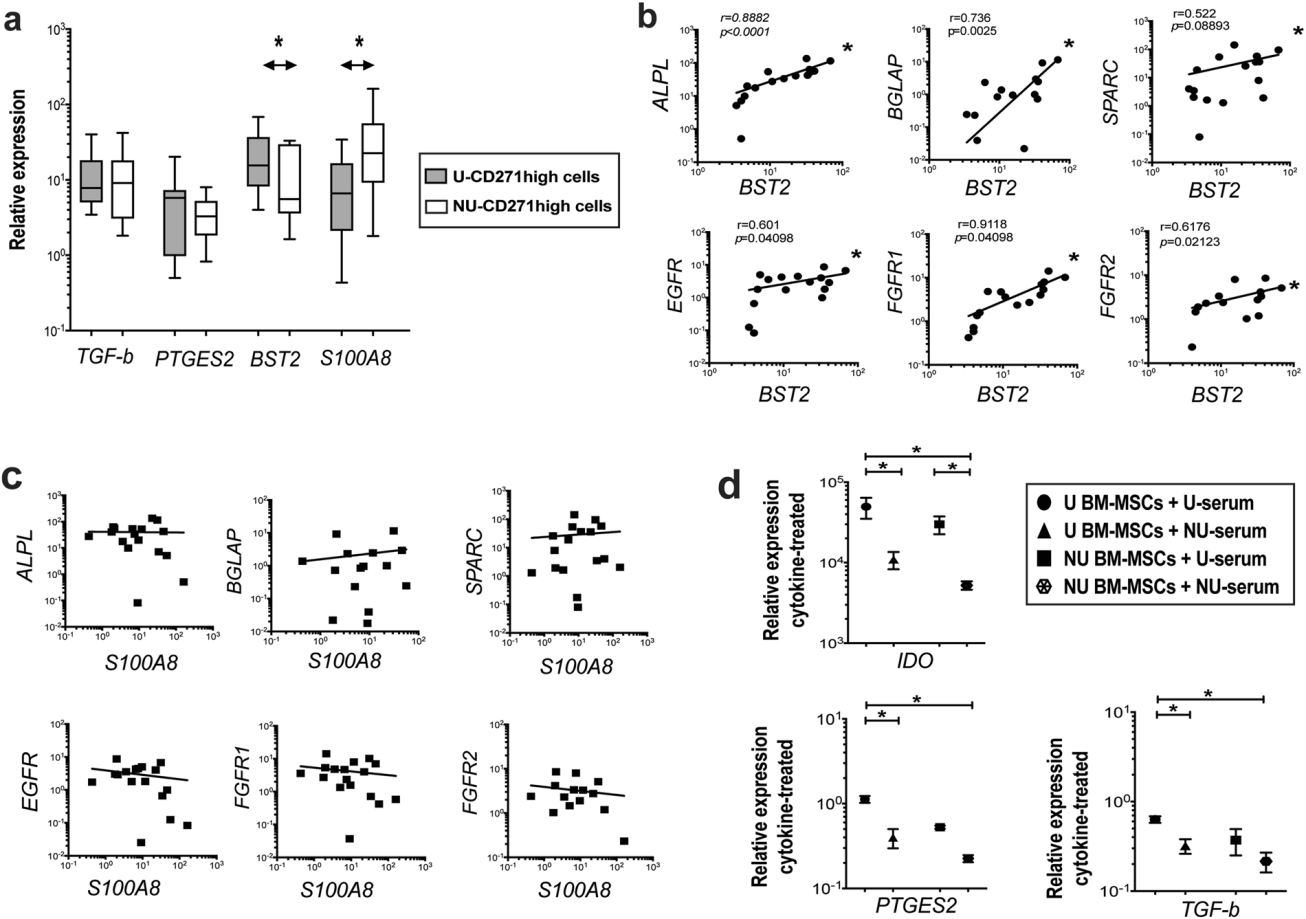
An altered immunoregulatory phenotype of NU BM-MSCs. (**a**) The figure shows the median of relative gene expression of immunoregulatory markers. The Mann-Whitney test was used to compare uncultured isolated NU-CD271^high^ CD45^low^ cells (n = 8) and U-CD271^high^ CD45^low^ cells (n = 9). (**b**) The plots show the correlation between the transcript levels of *BST2* versus those of osteogenic and growth factor receptors in CD271^high^ CD45^low^ cells. Spearman r test was used for the correlation (n = 17). (**c**) The plots show the correlation between the transcript levels of *S100A8* versus those of osteogenic and growth factor receptors in CD271^high^ CD45^low^ cells. Spearman r test was used for the correlation (n = 17). (**d**) The figure presents the mean of relative gene expression of *IDO, PTGES2* and *TGF-β1* when passage-zero BM-MSCs were treated with a mixture of IFN-γ, TNF-α, and IL-1 in U-serum or NU-serum supplemented cultures. The Kruskal-Wallis test was used to compare the groups (n = 3).

To assess the immunoregulatory potential of NU BM-MSCs, markers for immunosuppression and other new immunoregulation-related molecules were measured. Comparing uncultured NU- and U-CD271^high^ CD45^low^ cells, immunosuppressive *TGF-β1* and *PTGES2* transcripts were found similar (Fig. 3a). These data indicated no difference in the basal levels of these immunosuppressive mediators between U- and NU-MSCs. Notably, fewer transcripts of an immunoregulation-related marker, *BST2* were detected for uncultured NU-CD271^high^ CD45^low^ cells compared to U cells (p = 0.019, Fig. 3a and Table 2). In contrast, higher transcript levels of another immunoregulation-related molecule *S100A8* (p = 0.022, Fig. 3a and Table 2) were detected for NU-CD271^high^ CD45^low^ cells than U cells. Interestingly, the *BST2* transcript levels were positively correlated with those of *ALPL, BGLAP and SPARC, EGFR, FGFR1 & FGFR2* (p < 0.001, p = 0.003, p = 0.034, p = 0.012, p = 0.012, p = 0.021, respectively, Fig. 3b). However, *S100A8* transcript levels did not correlate with these markers (Fig. 3c). These findings indicated altered immunoregulatory markers of NU-MSCs with verification of BST2 link to osteogenic and proliferation of BM-MSCs.

The expression of immunosuppressive markers was further evaluated following treating passage-zero NU BM-MSCs with a mixture of IFN-γ, TNF-α, IL-1 and IL-17 in serum-supplemented cultures. Noteworthy, cytokine-treated NU BM-MSCs had lower *IDO, TGF-β1* and *PGES2* expression than U BM-MSCs in their matched serum cultures (p = 0.001, p = 0.005, p = 0.001, respectively Fig. 3d). Furthermore, U BM-MSCs were found to express fewer transcripts of *IDO*, *TGF-β1*, and *PGES2* in NU-serum cultures than U-serum cultures (p = 0.019, p = 0.019, p = 0.058, respectively Fig. 3d) suggesting an additional effect of NU-serum. Together, NU BM-MSCs demonstrated low expression levels of cytokine-induced immunosuppressive markers and particularly in NU-serum cultures suggesting both cellular and systemic-related causes.

### U and NU BM-MSCs had similar functions when culture-expanded

**Figure 4:**
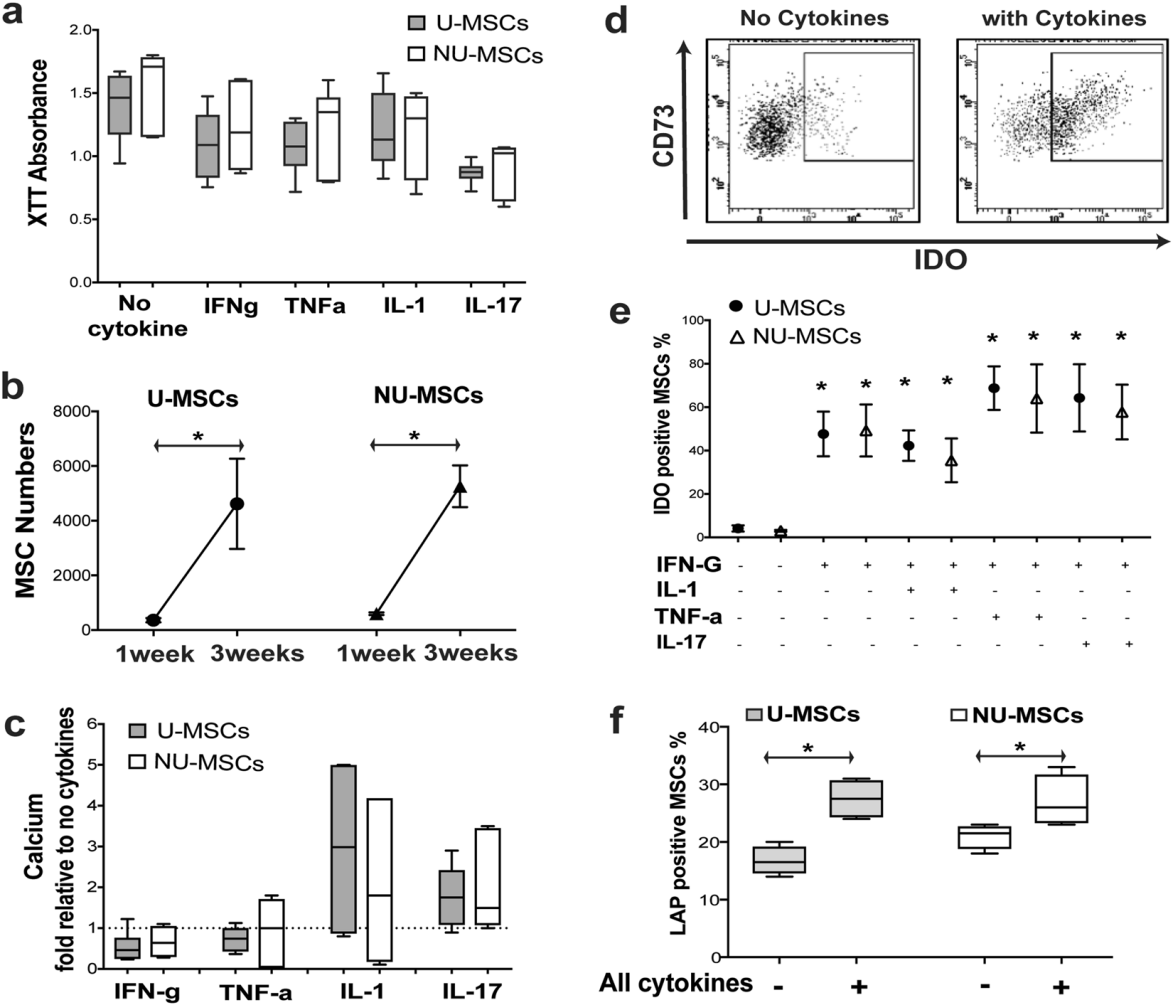
The comparison of culture-expanded NU-MSC and U-MSC functions. (**a**) The Whiskers plot shows the median of XTT absorbance (proliferation) for culture-expanded MSCs + /− treatment of inflammatory cytokines. The Mann-Whitney test was used to compare U-MSCs (n = 6) & NU-MSCs (n = 4). (**b**) The figure shows the mean (with the standard of the mean) of the MSC numbers released from scaffolds. The paired t-test was used for comparing 1-week and 3-week cultures and unpaired t-test for comparing U- and NU-MSCs (n = 3). (**c**) The Whiskers plot presents the median of calcium levels for culture-expanded MSCs following 3 weeks of culture in an osteogenic medium. The Mann-Whitney test was used to compare U-MSCs (n = 5) and NU-MSCs (n = 3). (**d**) The flow-cytometry plots show the gating of intracellular IDO expression by culture-expanded MSCs. (**e**) The mean (with the standard of the mean) of the percentages of positive-expressing IDO in culture-expanded MSCs. The paired t-test was used to compare donor-matched no-cytokine with cytokines groups. The unpaired t-test was used to compare U-MSCs and NU-MSCs (n = 4). (**f**) The median of the percentages of positive-expressing LAP in culture-expanded MSCs. The paired t-test was used to compare donor-matched no-cytokine with cytokines groups. The unpaired t-test was used to compare U-MSCs and NU-MSCs (n = 4).

The uncultured and minimally-cultured BM-MSCs showed reduced proliferative capacity, lower expression of osteogenic markers with altered expression immunoregulatory markers. To test if these detected changes have a functional impact on BM-MSCs, culture expansion of these progenitors was performed to get enough cells for the functional assays. The culture-expanded U- and NU BM-MSCs with/without cytokines had no significant differences in the XTT absorbance (Fig. 4a) indicating similar *in vitro* proliferative capacity. To further confirm these data, BM-MSCs were loaded on scaffolds, and the numbers of NU- and U-MSCs (counted as CD45^−^ CD73^+^ CD90^+^ CD105^+^ cells using flow-cytometry,^48^) were similarly increased after 3-week cultures relative to 1-week cultures (p = 0.007 and p = 0.003 respectively, Fig. 4b) confirming similar proliferation for culture-expanded NU-MSCs and U-MSCs.

For testing the osteogenic differentiation, culture-expanded BM-MSCs were cultured for 3 weeks in osteogenic medium with/without cytokine treatment then the calcium deposition levels were measured. While IL-1 and IL-17 (but not IFN-γ, and TNF-α) seemed to induce calcium levels in differentiating NU and U BM-MSCs (Fig. 4c), measured calcium levels were comparable for NU and U BM-MSCs either with or without cytokine treatments (Fig. 4c). These data demonstrated the similar osteogenic capacity of culture-expanded NU and U BM-MSCs.

For immunosuppression, cytokine-treated culture-expanded MSCs were assessed for the intracellular IDO levels using flow-cytometry (Fig. 4d). As expected, the IDO levels were similarly induced when MSCs were treated by IFN-γ alone or combined with TNF-α, IL-1 or IL-17 (all *p* < 0.001, Fig. 4e). Importantly, these induced IDO levels were similar for NU- and U-MSCs (Fig. 4e). Also, the percentage of culture-expanded NU- and U-MSCs expressing LAP (surface TGF-β1) were similarly increased after cytokine treatments (p = 0.040 p = 0.006, respectively, Fig. 4f). These results indicated comparable immunosuppressive functions of culture-expanded NU- and U-MSCs.

### The expression of cytokine receptors on BM-MSCs and comparable serum cytokine levels during early fracture healing

**Figure 5:**
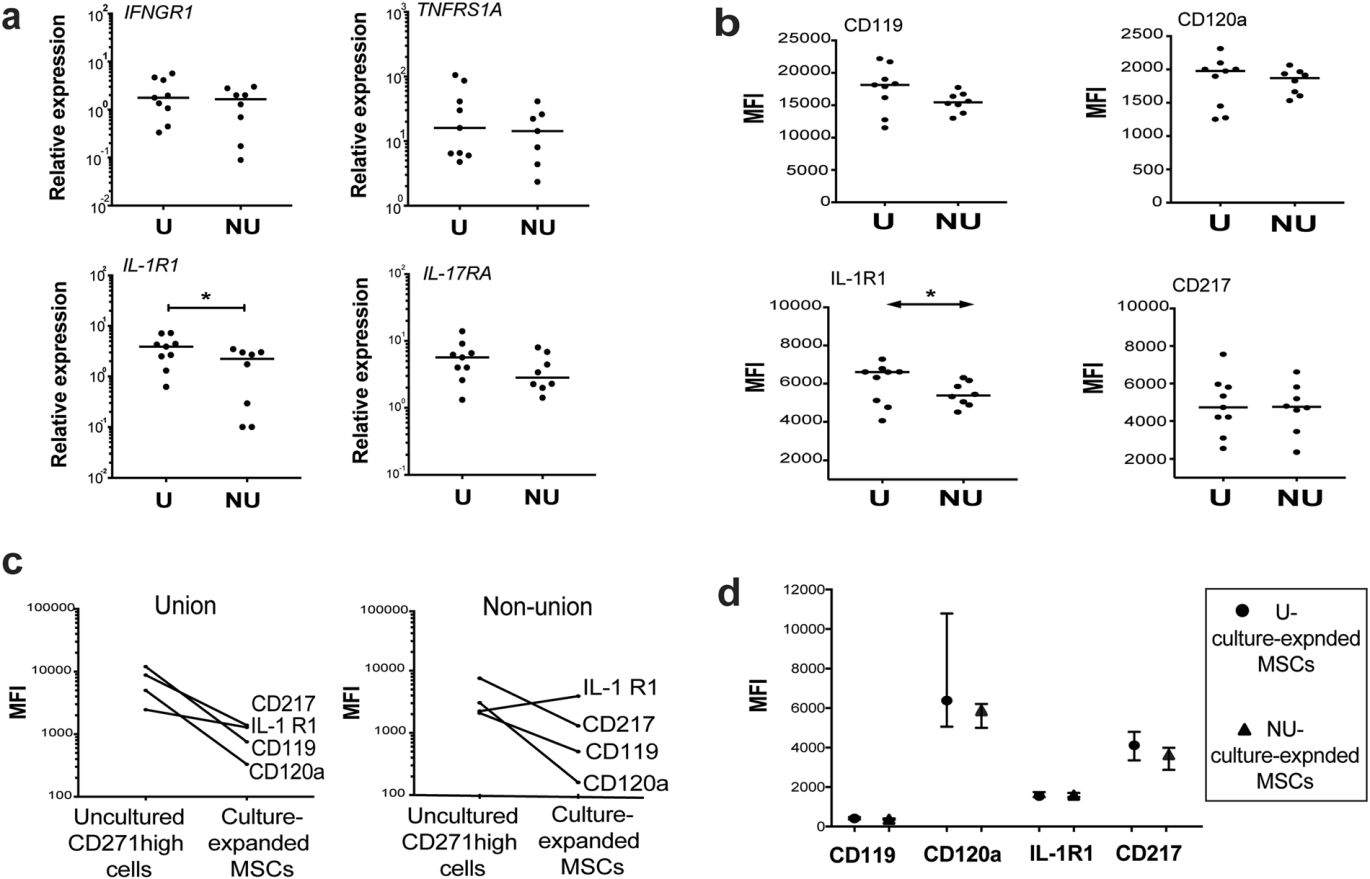
The measurements of cytokine receptor expression for BM-MSCs. (**a**) The scatter dot plots show the median of relative gene expression of cytokine receptors. The unpaired t-test was used to compare uncultured isolated NU-CD271^high^ CD45^low^ cells (n = 8) and U-CD271^high^ CD45^low^ cells (n = 9) for all cytokine receptors except for TNFR1, in which the Mann-Whitney test was used. (**b**) The scatter dot plots present the median of surface expression levels of cytokine receptors. The unpaired t-test was used to compare uncultured NU-CD271^high^ CD45^low^ cells and U-CD271^high^ CD45^low^ cells (n = 8 and 9 respectively) for all cytokine receptors except for CD217 (IL-17RA), in which the Mann-Whitney test was used. (**c**) The figure demonstrates the changes in cytokine receptor levels between donor-matched uncultured CD271^high^ CD45^low^ cells and culture-expanded MSCs. (**d**) The figure presents a comparison of cytokine receptor levels between culture-expanded NU-MSCs (n = 3) and U-MSCs (n = 6). The Mann-Whitney test was used for the comparison.

As the minimal cultures data indicated that NU BM-MSCs were less immunosuppressive when cytokine-treated compared to U-MSCs, we next tested if the cytokine receptor levels could be a contributing factor. Interestingly, the uncultured NU-CD271^high^ CD45^low^ cells expressed fewer transcripts of *IL-1R1* than U-CD271^high^ CD45^low^ cells (p = 0.005, Fig. 5a). However, no significant differences were detected for other cytokine receptor transcripts (Fig. 5a). Consistently, the surface protein expression of the cytokine receptors was similar for uncultured NU-and U-CD271^high^ CD45^low^ cells except for IL-1R1, which were less expressed on the surface of NU-CD271^high^ CD45^low^ cells (p = 0.049, Fig. 5b).

To explore why contrarily culture-expanded BM-MSCs have similar immunosuppressive functions, the surface cytokine receptor expressions were compared between donor-matched culture-expanded MSCs and uncultured CD271^high^ CD45^low^ cells. The data showed various changes in all receptors surface expression (mainly a decrease after culture-expansion) in both U-MSCs and NU-MSCs (Fig. 5c). Furthermore, comparable cytokine receptor levels were noted between culture-expanded NU- and U-MSCs (Fig. 5d) verifying changes of MSC surface phenotype during *in vitro* culture-expansion.

**Figure 6:**
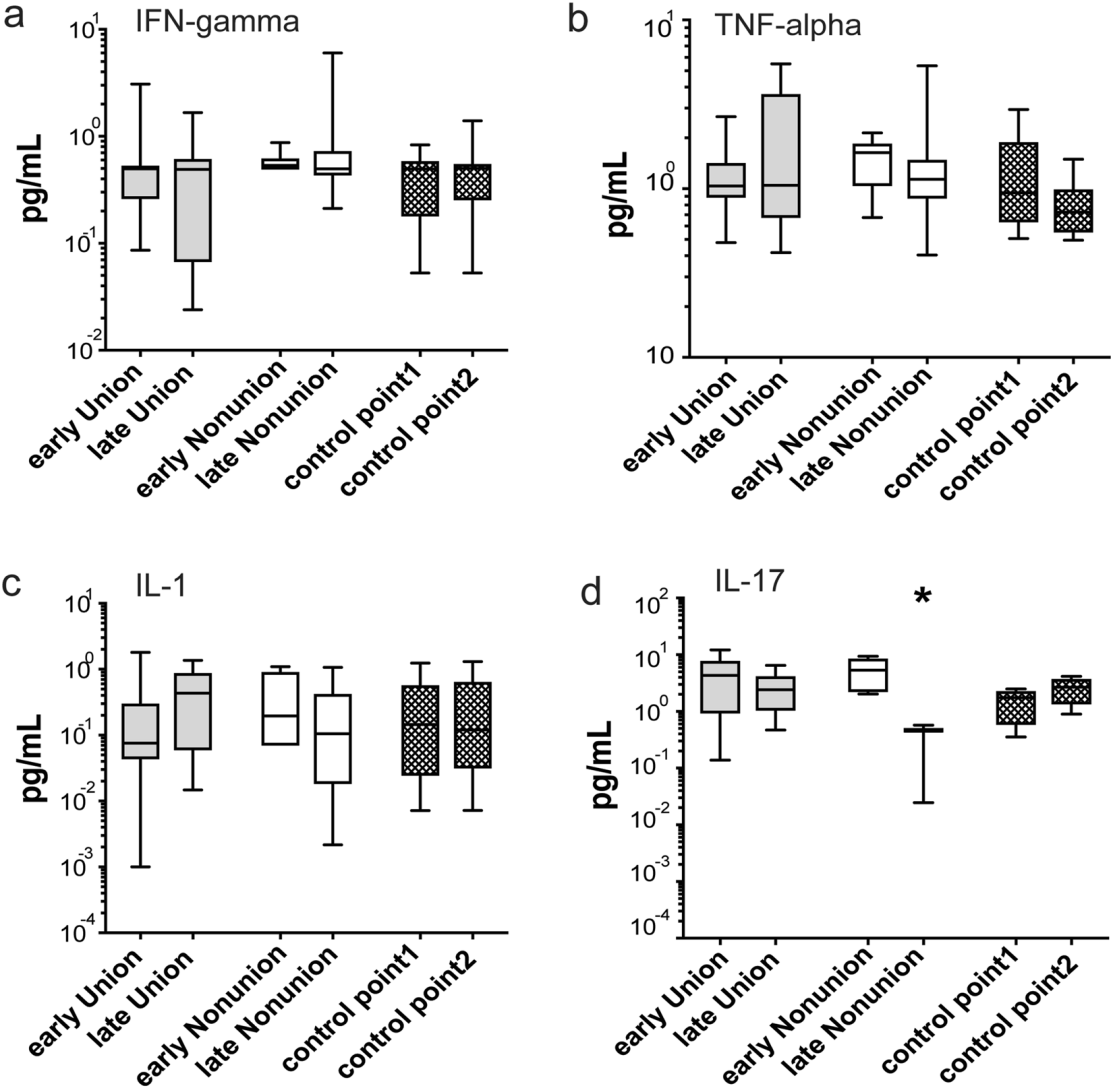
The measurements of serum cytokine levels. The figures show the median of serum levels of IFN-γ, TNF-α, IL-1 and IL-17 (**a–d** respectively) during a week of fracture (early healing), at or after 6 months of fracture (late healing) and healthy control levels (two-time points, 6-month apart). The Kruskal-Wallis test was used to compare levels of IFN-γ, TNF-α, IL-1 and IL-17 between U-serum (early healing: n = 9, 12, 9 and 13 respectively, late healing n = 9, 12, 9 and 11 respectively), NU-serum (early healing n = 5, late healing n = 9, 12, 9 and 6 respectively) and control serum (both time points: n = 8, 11, 7 and 5 respectively).

We further aimed to understand if serum cytokines levels are linked to the decreased proliferative potential and altered immunosuppressive phenotype of native BM-MSCs. Therefore, the levels of immunosuppression-related cytokines were measured in serum of fracture patients relative to control levels. The ELISA data showed that IFN-γ, TNF-α, and IL-1 levels were not different between U-sera, NU-sera and control sera (Fig. 6a–c). These findings indicated no systemic changes in these cytokine levels during fracture healing. Noteworthy, the measured IL-17 levels were alike for U and NU fractures during early healing. However, significant lower IL-17 levels were detected for late healing NU-serum than healed U-serum and control levels (p < 0.001 and  p < 0.001, respectively, Fig. 6d). Collectively, apart from IL-17 measured in NU serum at late healing, comparable serum cytokine levels were noted for NU and U.

## Discussion

The application of autologous BM to boost the healing of non-united fractures as a source of osteoprogenitors has gained lately a great popularity^49–52^. However, the quality of these BM-MSCs is poorly understood. In this study, we comprehensively compared various functional potentials of BM-MSCs from NU and U fracture patients utilising multiple assays and at different cell culture conditions. The functional potential of uncultured CD271^high^ CD45^low^ cells and minimal MSC cultures have shown significant differences between NU and U BM-MSCs, implying defective multifunction of NU BM-MSC. However, the functions of culture-expanded MSCs were similar. This masking of cell differences within the culture-expanded cells is most likely due to *in vitro*-related changes in cell phenotype such as those changes we detected for the expression of the surface cytokine receptors. Similarly, adipose-derived MSCs showed significant variations in cell cycle and functional transcripts during culture-expansion^53^. We think that foetal calf serum (FCS) could be a contributing factor to phenotype changes, particularly with previous research has shown that human serum, unlike FCS, can maintain the MSC genomic patterns^54^.

With regards to the proliferative capacity, we identified significant lower transcripts levels of *EGF* and *FGF* receptors for uncultured NU CD271^high^ CD45^low^ cells than U cells. These findings implied a weak response to EGF and FGF, which are known to be essential for the MSC proliferation and survival, particularly in fractures^55–58^. Also, a role for EGF in fracture healing has been suggested when its serum levels were found increased in patients with a brain injury combined with a limb fracture than those having brain injury only^59^. In agreement with our findings, a previous study has reported downregulation of FGF-R2 gene expression in human non-union osteoblast cultures^35^. In addition to the MSC response to growth factors, we noted an inadequate proliferation of passage-zero BM-MSCs in cultures supplemented with NU-serum compared to U-serum cultures inferring lower growth inducers in NU-serum. Our group previously showed that the proliferative capacity of BM-MSCs from fracture patients was positively correlated with serum growth factor levels, such as platelet-derived growth factors (PDGFs)^55^. In total, it seems that the proliferation of BM-MSCs in non-union patients could be defective due to both intrinsic cellular and extrinsic microenvironmental factors. Such complicated alterations of the NU BM-MSC proliferation could be potentially compensated for by applying adequate numbers of BM-MSCs as suggested for therapies of non-united fractures^51^.

We detected significantly lower osteogenic marker levels in uncultured NU BM- CD271^high^ CD45^low^ cells despite the osteogenic ability shown for culture-expanded cells in this study and previous work^41^. In NU-serum supplemented cultures, all osteogenic genes, except *ALPL* showed comparable levels versus U-cultures. These observations could be explained by the positive osteoinductive role of human serum effect on progenitor osteogenesis as reported before^60^. Together, unlike culture-expanded MSCs, our data uniquely suggested a lower osteogenic potential of native NU BM-MSCs compared to U BM-MSCs. This apparent osteogenic defect could be probably improved by co-application of autologous serum growth factors, e.g., concentrated platelet lysate products. Consistent with our findings of low osteogenic potential of BM-MSCs in NU patients, the serum levels of several osteogenic markers have been shown to decrease in NU fractures. Kurdy *et al*. and Emami *et al*. detected lower levels of ALP in patients with delayed healed tibial fractures than normal healers^61,62^. Additionally, other groups reported reduced levels or delayed induction of osteocalcin levels in NU than U fractures^63–65^. Furthermore, the concentrations of C-terminal cross-linking telopeptide of type I collagen measured in the serum of NU patients were significantly less than those in U group^66^.

Although early inflammatory response is critical for the healing of fractures and bone loss, unbalanced inflammation marked by high levels of cytokines, TNF-α, IFN- γ and IL-1 TNF-α in fracture hematoma or callus could hinder a successful bone regeneration and lead to complicated healing (or non-union)^11,20,67–69^. Schmidt-Bleek *et al*. also have reported that higher relative expression of the pro-inflammatory cytokines together with a higher percentage of cytotoxic T cells in the hematoma of delayed healing than those of normal healed fracture^70^. Several studies reported that MSCs could drive immunosuppressive response as a part of their role in bone healing e.g., decreasing cytokines levels in association with enhancing the osteogenesis in fractures or osteoporosis models^24–26^. Uniquely, we showed here that NU BM-MSCs had fewer transcripts of *IDO, PTGES2* and *TGF*-β1 when cytokine-treated than those of U BM-MSCs. Furthermore, low expression of IL-1 receptors in NU BM-MSCs might also suggest less response to such a priming cytokine. Altogether, our findings showed that NU BM-MSCs could have less immunosuppressive potential that could negatively affect normal bone healing.

In addition to immunosuppressive markers, other markers related to BM-MSCs immunoregulatory potential have been recently described. An example is the detection of high levels of BST2 expression in specific clones of BM-MSCs that secrete an immunoregulatory cytokine, IL-6^29^. We also have reported that S100A8 was highly expressed on hematopoietic cell-depleted cancellous bone populations that are capable of inhibition of T cell proliferation^31^. Our novel data here showed that NU BM-MSCs expressed lower *BST2* transcripts, but higher *S100A8* transcripts than U BM-MSCs. Furthermore, *BTS2*, but not *S100A8* transcript levels were positively correlated with the osteogenic markers and growth receptor levels, confirming previous results of BST2 involvement in the MSC osteogenic differentiation^30^. The high *S100A8* transcript levels in NU BM-MSCs could be linked to dysregulated bone remodelling as S100A8 has been shown as a contributor in the remodelling process^71^.

We detected significantly lower levels of IL-1 receptors consistently at protein and gene expression levels in NU BM-MSCs, also validating our PCR data. Growing evidence has indicated that IL-1 could have an individual or additive role to IFN-γ in enhancing MSC-mediated immunosuppression^72,73^. Additionally, IL-1 was shown to enhance osteogenesis, particularly for periodontal ligament stem cells^74^. Therefore, the functional impact of low expression of IL-1R on bone healing would be interesting for further investigations.

We measured the serum levels of cytokines that are known to prime BM-MSCs for immunosuppression. Our data showed no differences in serum levels of IFN-γ, TNF-α, IL-1, and IL-17 between patients or healthy non-fracture controls. Unlike serum, high levels of TNF-α were noted in fracture hematoma initially, then these levels were decreased with repair, but were highly expressed again during remodelling^75–77^. Also, IFN-γ expression levels were reported to be increased at hematoma^78^. Exceptionally, the IL-17 levels in NU-sera were significantly lower than U- and control serum in late healing. IL-17 was detected in soft callus during bone healing^79^ and shown to have a complicated role during remodelling being supportive of osteoblastic bone formation but also involved in the stimulation of osteoclasts formation^80–82^. Our data showing lower IL-17 levels in NU sera than controls might suggest a relation to the disturbance in the bone remodelling process. The expression levels of several serum cytokines, growth factors, and other inflammation-related molecules in NU fracture patients were thoroughly assessed in many previous studies. However, and up to our knowledge, we are the first to compare serum cytokines, IFN-γ, TNF-α, IL-1, and IL-17 between NU, U patients, and healthy controls and found comparable levels. Likewise, another study showed that serum levels of IL-8, which is linked to human MSC migration^83^ were not different between NU fractures and controls^84^. Furthermore, serum levels of other cytokines linked to BM-MSC migration, such as Macrophage colony-stimulating factor (M-CSF) and SDF-1^85^, were found to be similar between patients with physiological fractures and those with impaired fracture healing^84,86^. In contrast, Mathieu *et al*. found higher serum levels of IL-6 and lower levels of soluble IL-6 receptors in NU patients compared to healthy individuals^84^. Interestingly, an *in vitro* work showed that high concentrations of IL-6 with low expression of soluble IL-6 receptors could inhibit MSC differentiation^87^.

In terms of growth factors, TGF-beta, PDGF, FGF, and IGF are critically important for differentiation and growth of MSCs^58,88^. In fractures, TGF-β1 serum levels were found peaked between 1 and 6 weeks after trauma in normal healers, but it lasted for a shorter time and lower levels in NU patients^89,90^. Additionally, reduced PDGF serum levels in NU fractures were noted compared to U fractures^84,90,91^. Similarly, lower serum levels of FGF-2 in paediatric NU patients than normal healers were reported^92^. Previous data also showed low and stable levels of serum insulin-like growth factor-1 (IGF-1) and its specific binding proteins in NU fractures^84,90,93^. While serum VEGF concentrations in NU patients were found comparable to those in patients with normal fracture healing in some studies^84,94^, others found that serum VEGF is increased when NU fracture is treated^95^. Compared to systemic changes in these growth factors, other growth factors seemed to have local changes. Serum BMP-2 and BMP-4 were below the detection level in both U and NU patients^90^. Another work demonstrated no significant difference in plasma levels of BMP-2, -4, -6, and -7 between patients with NU and those with normal fracture healing^96^. However, the expression levels of these BMPs were significantly lower when assessed within fibrous NU tissue relative to a standard healing callus^33,97^.

Additional serum factors that are related to skeletal tissue inflammation or BM-MSC immunomodulatory functions were also quantified by other groups. In one study, NU patients had significantly higher serum concentrations of metalloproteinase protein-1 (MMP-1) and MMP-8, which are linked to skeletal tissue inflammation^98^ compared with those with normal fracture healing^99^. S100A9 is also secreted by blood cells, and its high serum levels are linked to inflammation, particularly of musculoskeletal tissues^100^. Interestingly, serum S100A9 has been found elevated than normal in NU patients^101^, reflecting a local inflammation of NU tissues in which MSCs could be involved. Another work by Wang *et al*. demonstrated that Nitric oxide (NO), which is linked to MSC differentiation and bone remodelling^102^, had significantly lower levels in persistent NU patients relative to those having good healing progress^95^. Furthermore, proteomic analysis of serum showed that complement C6, C3 and C4, which their strongly activating phenotype is less favourable for MSC-related immunosuppression^85^, were up-regulated in the serum of NU patients compared to healthy controls^101^. Altogether, the systemic changes of various serum mediators could explain the lower proliferative and osteogenic potential as well as the altered immunomodulatory potential of the MSC pool in BM that we noted in our study and by others for NU fracture patients^103^.

Having NU BM-MSCs collected retrospectively was a limitation in our study. We could not prove that the altered immunoregulatory phenotype of these NU BM-MSCs (i.e. being less immunosuppressive, low BST-2 and IL-1R and high S100A8 expression) was consistently the same phenotype at the early phase of healing. Further research using a large panel of NU BM-MSCs prospectively collected within the early phase of fracture healing would be of great value to confirm our findings of the altered immunoregulatory phenotype. Such research could potentially help to introduce new predictive molecules for non-union fracture at the early phase and perhaps allowing earlier intervention with therapies to enhance bone repair.

In conclusion, our data of uncultured BM-MSCs indicated some interesting differences between union and non-union MSCs. These native NU BM-MSCs seemed to have a low proliferative capacity and osteogenic potential relative to U BM-MSCs. Additionally, our study is the first report of an altered immunoregulatory phenotype of NU BM-MSCs with these cells appeared to have reduced immunosuppressive potential and lower expression of BST2. Clinically, the quality of native uncultured autologous BM-MSCs used for enhancing bone regeneration should be considered. One may argue that the therapeutic value of autologous native BM-cells could be improved with growth adjuvants as well as placing adequate numbers of these BM-MSCs *in situ* to compensate for potentially low performance.

The functions of culture-expanded NU and U BM-MSCs were comparable most probably due to changes in cell phenotype. Therefore, to assess the MSC functions to a close picture as possible to *in vivo* cell performance, functional experiments would need to be optimised on minimally-cultured MSCs and in the presence of human serum. In such an experimental approach, future analysis of the functional impact of low IL-1 receptor levels in NU BM-MSCs could potentially help to improve cell-based bone regenerative therapies. Additionally, further work would be required to confirm a specific immunoregulatory phenotype of NU BM-MSCs at the early healing phase, which could potentially help to predict the risk of non-union fractures.

## Methods

### Ethics statement

Informed written consent was obtained from all the study participants before the samples were collected, and research was carried out in accordance with the Helsinki Declaration of ethics. The consents and sample collection (blood and bone marrow samples used to extract MSCs) for this study were under ethical approval with NREC number, 06/Q1206/127, National Research Ethics Committee Yorkshire and Humber–Leeds East. All study experiments were conducted according to the appropriate guidelines and regulations.

### Participants and samples

Overall seventy-one participants were included in the study (Table 2**)**. All patients had long bone fractures (femur, tibia, humerus). NU was defined by the absence of radiological features of fracture healing (lack of callus formation in at least 3 cortices) either on plane radiographs or computed tomography scans after 9 months from fracture fixation and with ongoing pain at the NU site during ambulation. Exclusion criteria were children, cancer, diabetes, bone metabolic diseases, inflammatory/immune disorders and intake of drugs with a negative impact on fracture healing (i.e. NSAIDs). BM samples (15 ml) were aspirated from the anterior iliac crest as previously described^104^; within one week following injury for U patients, and after diagnosis (>9 months) for NU patients. Peripheral venous blood samples (12 ml) were collected (from the patients) within one week from the date of fracture (early healing phase) or after fracture had united (4–6 months from injury) for U fractures and at the time of NU diagnosis. Serum from healthy individuals who did not have any bone fractures was used as control (twice, 6-month apart). Comparing the age of three groups showed no significant difference (Kruskal-Wallis test, *p* = 0.497).

**Table 2:** The study participants.

	Non-union patients	Union patients	Healthy volunteers
Age median	49 years	44 years	42 years
Age range	18–76 years	20–75 years	23–60 years
Male	16	23	7
Female	6	14	5
Total	22	37	12
Samples	BM aspirates and Blood	BM aspirates and Blood	Blood

The age and gender for the patients and healthy volunteers included in the study with sample types.n

### Isolation of uncultured BM cells and real-time PCR

BM-cells were processed for erythrocyte lysis as reported before^105^ then labelled with CD45 and CD271 antibodies (Miltenyi) and live/dead cell markers; Calcein violet/Aqua (ThermoFisher) for cell sorting. Uncultured CD271^high^ CD45^low^ cells as surrogates of MSCs^43–46^ and control hematopoietic lineage cells (HLCs, CD271^negative^ CD45^positive^ cells) were isolated using Fluorescence-activated cell sorting (FACS, Becton-Dickinson).

The quantitative real-time PCR using TaqMan probes (ThermoFisher, Table 1) was conducted for measuring the relative gene expression levels of chosen markers. RNA extraction was performed as per manufacturer recommendation (Norgen Biotek). Extracted RNA samples were processed for reverse transcription and then pre-amplification using kits (Fluidigm) as recommended. Finally, the gene expression assays were performed using 48.48 chip/Integrated Fluid Circuit (IFC) and using the Biomark™ HD system (Fluidigm). The marker transcript levels were calculated relative to that of a housekeeping gene, Hypoxanthine Phosphoribosyl transferase 1 (HPRT1).

### BM-MSC cultures

BM-cells were cultured till plastic-adherent MSCs become confluent then lysed and analysed without passage (passage-zero cells). These cultures were maintained in DMEM medium (Sigma) supplemented with 10% pooled U- or NU-serum (from five donors and collected within one week of fracture). Some cultures were treated with cytokines (20 ng/ml IFN-γ, 20 ng/ml TNF-α, 10 ng/ml IL-1 and 100 ng/ml IL-17) as per previous studies^106–108^. For the culture-expansion, BM-cells were maintained in the StemMACS MSC Expansion medium (Miltenyi Biotec) and plastic-adherent MSCs were passaged then analysed at passage 3–4.

### Proliferation

The proliferation of culture-expanded BM-MSCs was assessed using the XTT colorimetric assay kit (Merck). For testing the growth of culture-expanded BM-MSCs on Orthoss collagen^®^ scaffold (Geistlich), 10 × 10^4^ cells were loaded on 60 mm^2^ pieces of the scaffolds for 3 hours before maintaining in cultures. The loaded scaffolds were digested after 1 and 3 weeks of culture using collagenase (Stem cell technologies). The released cells were then characterised and counted by flow-cytometry as shown previously^105,109^. For passage-zero BM-MSCs in serum-supplemented cultures, the housekeeping 18 S expression was evaluated, indicating cell quantities as reported before^110^.

### Osteogenesis and immunosuppression assays

The culture-expanded BM-MSCs were assessed for the osteogenesis by measuring the calcium levels after 3 weeks in osteogenic cultures^105^. The calcium levels were measured using a colorimetric kit (Sentinel)^105,99,78,78^.

The immunosuppressive capacity of culture-expanded BM-MSCs was tested following treatment for 5 days with cytokines. The cells were then processed for measuring the intracellular IDO and surface TGF-β1 latency-associated peptide (LAP) levels using specific conjugated monoclonal antibodies (ThermoFisher) for flow-cytometer as demonstrated in previous studies^111–113^. For passage-zero cultured BM-MSCs that were cytokine-treated, the expression levels of *IDO*, *PGE2* and *TGF-ß* transcripts was evaluated indicating the MSC immunosuppressive potential.

### Surface cytokine receptor measurement

Flow-cytometry was used to measure the cytokine receptor levels on the surface of uncultured NU and U CD271^high^ CD45^low^ cells (and to validate PCR data) or on culture-expanded NU and U MSCs. The flow-cytometry conjugated monoclonal antibodies against IFN-γ receptor 1 (CD119), TNF-α receptor 1 (CD120a), (both from Miltenyi Biotec), IL-17 receptor A (CD217), (R&D systems) and IL-1β receptor 1 (BD Biosciences) were used for these assays.

### Serum cytokine measurements

The blood samples were processed by centrifugation (2000g for 15 minutes) to extract the serum, as previously described^114^. The separated sera were analysed for the IFN-γ, TNF-α, IL-1 and IL-17 levels using the hypersensitive ELISA kits (R&D systems) as recommended by the manufacturer. The optical density values were acquired using MULTISCAN EX reader and analysed with Ascent software (Thermo electron corporation).

### Statistical analysis

The statistical analysis and figures’ preparation were performed using GraphPad Prism 7 software. The Shapiro-Wilk normality test was applied to choose the comparative tests between groups. These comparative tests were also applied according to the group numbers and being paired or non-paired; all indicated in figure legends. Correlations were tested using the Spearman rho test. Any difference between the groups was considered as statistically significant when the p value < 0.05.

## Supplementary information

Dataset 1

## Data Availability

The datasets supporting the conclusions of this study are included within the article and its additional supporting file.
